# Supplementary material for: Turnover of SARS-CoV-2 Lineages Shaped the Pandemic and Enabled the Emergence of New Variants in the State of Rio de Janeiro, Brazil
Source: Viruses. 2021 Oct 7;13(10):2013. doi: 10.3390/v13102013 (PMC8537965; doi:10.3390/v13102013)
Supplement: Supplementary file 1 [file viruses-13-02013-s001.zip › Francisco_Junior_et_al_2021_Supplementary_Material.pdf]

Supplementary Material

Turnover of SARS-CoV-2 lineages shaped the pandemic and enabled the emergence of new variants in the state of Rio de Janeiro, Brazil

Ronaldo da Silva Francisco Junior[1#], Alessandra P Lamarca[1#], Luiz G P de Almeida[1], Liliane Cavalcante[1], Douglas Terra Machado[1], Yasmmin Martins[1], Otávio Brustolini[1], Alexandra L Gerber[1], Ana Paula de C Guimarães[1], Reinaldo Bellini Gonçalves[1], Cassia Alves[2], Diana Mariani[2], Thais Felix Cruz[2], Isabelle Vasconcellos de Souza[3], Erika Martins de Carvalho[3], Mario Sergio Ribeiro[4], Silvia Carvalho[4], Flávio Dias da Silva[5], Marcio Henrique de Oliveira Garcia[5], Leandro Magalhães de Souza[6], Cristiane Gomes Da Silva[6], Caio Luiz Pereira Ribeiro[5], Andréa Cony Cavalcanti[6], Claudia Maria Braga de Mello[4], Cláudio J. Struchiner[7], Amilcar Tanuri[2a], Ana Tereza R Vasconcelos[1a]*

[#]Both authors contributed equally

[a]Both authors coordinated this work

*Corresponding author : atrv@lncc.br

[1] Laboratório de Bioinformática, Laboratório Nacional de Computação Científica, Petrópolis, Brazil.

[2] Departamento de Genética, Instituto de Biologia, Universidade Federal do Rio de Janeiro, Rio de Janeiro, Brazil.

[3] Unidades de Apoio ao Diagnóstico da Covid-19, Rio de Janeiro, Brazil.

[4] Secretaria Estadual de Saúde do Rio de Janeiro, Rio de Janeiro, Brazil.

[5] Secretaria Municipal de Saúde Rio de Janeiro, Rio de Janeiro, Brazil

[6] Laboratório Central de Saúde Pública Noel Nutels, Rio de Janeiro, Brazil.

[7] Fundação Getúlio Vargas, Rio de Janeiro, Brazil

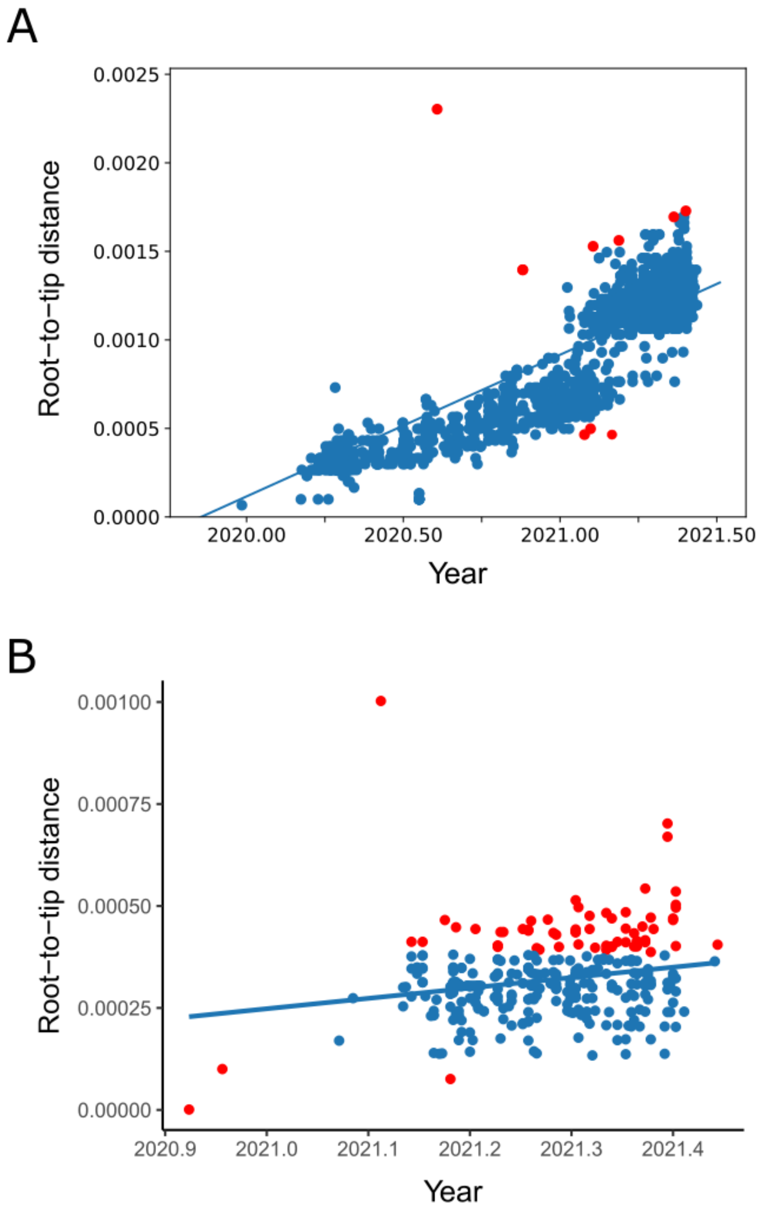

Figure S1. Correlation between root-to-tip distance and tip sampling date. A) Root to tip distance for the phylogenetic tree of genome sequences from Rio de Janeiro (Figure 1A). In red, the tips ignored by TreeTime to rescale the tree into dates. B) Root to tip distance for the P.1.2 tree reconstructed using maximum likelihood. In red, the tips that were not contained within the 95% confidence interval and were removed for the bayesian analysis.

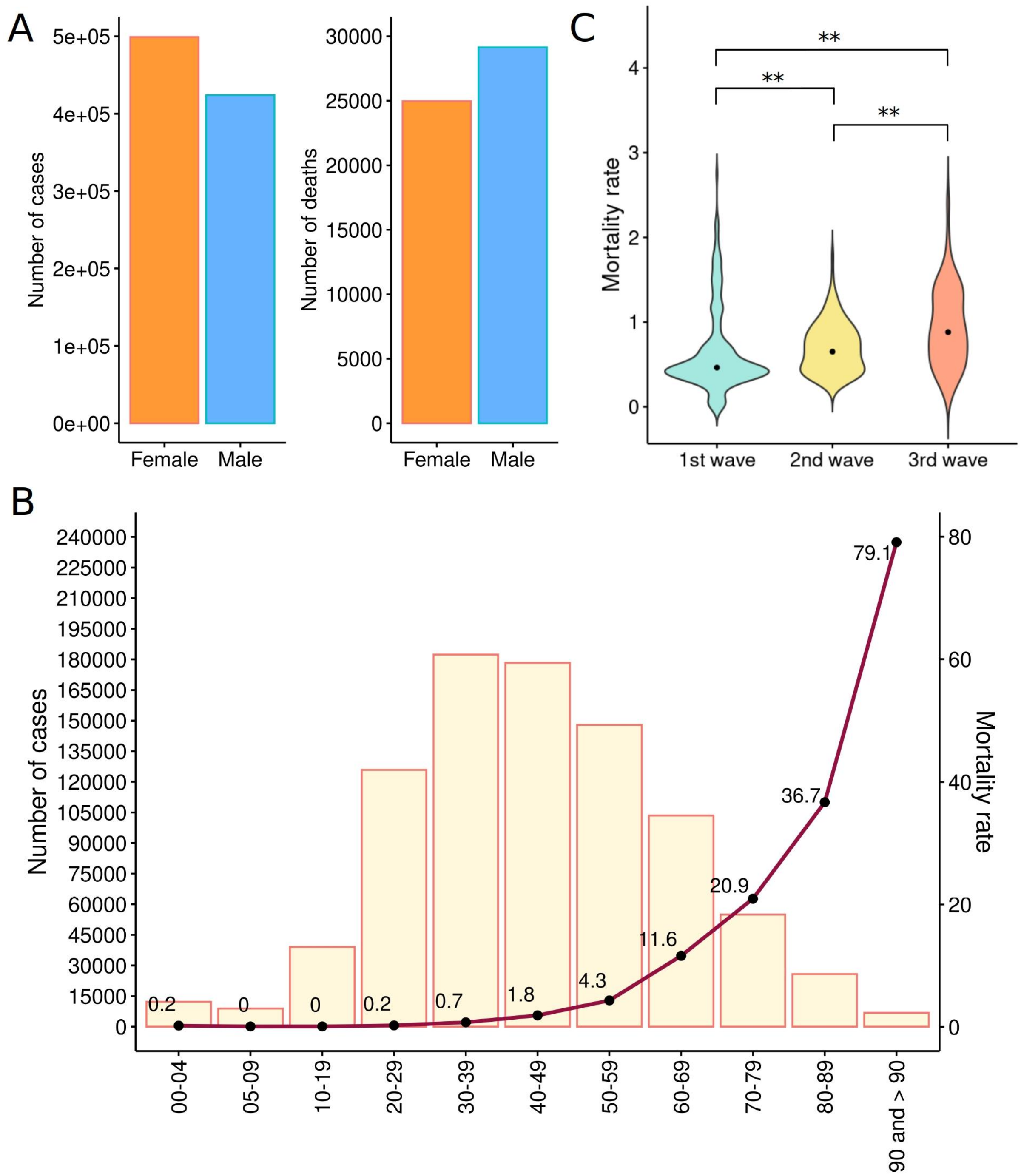

Figure S2. **Epidemiological description of the number of cases, deaths and the lethality rate.** A) Number of cases and deaths among females and males. B) Number of cases within age groups. Red line indicates the mortality rate within the age group. C) Differential mortality rate between the three phases using the Wilcoxon pairwise test with Benjamini-Hochberg p-value correction. Asterisk indicated statistical significant differences. The p-values are indicated as $p < 0.05$ (*) and $p < 0.01$ (**).

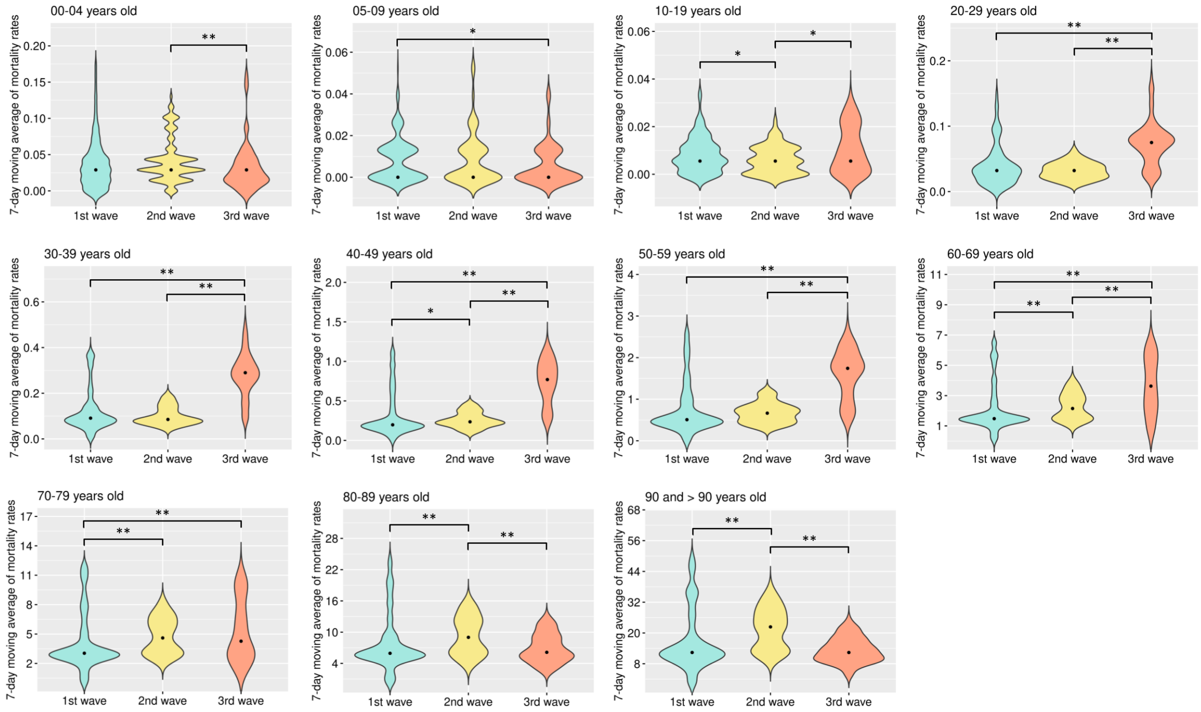

Figure S3. **Comparison of mortality rate within age group among the three phases.** Different ages were compared with a 7-day moving average mortality rate. The Wilcoxon pairwise test was used to compare the three phases in each age group, followed by correction by Benjamini-Hochberg. The p-values are indicated as $p < 0.05$ (*) and $p < 0.01$ (**).

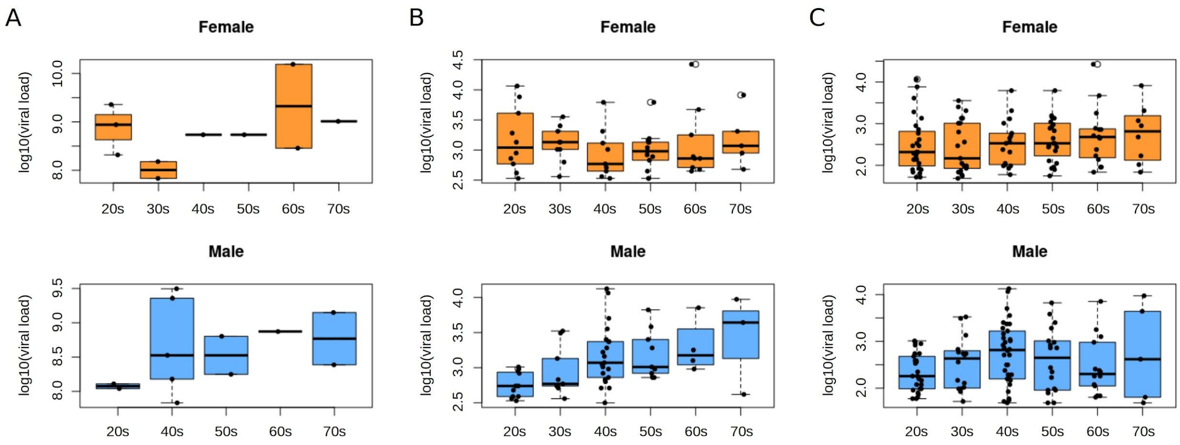

Figure S4. Comparison between the distribution of the relative quantification of the SARS-CoV-2 viral loads (RQVL) within an age group of 1,119 samples. Gamma-generalized linear model showed a significant difference in the RQVL among males age groups for both 2% (A), 10% (B) and 20% (C) of the population.

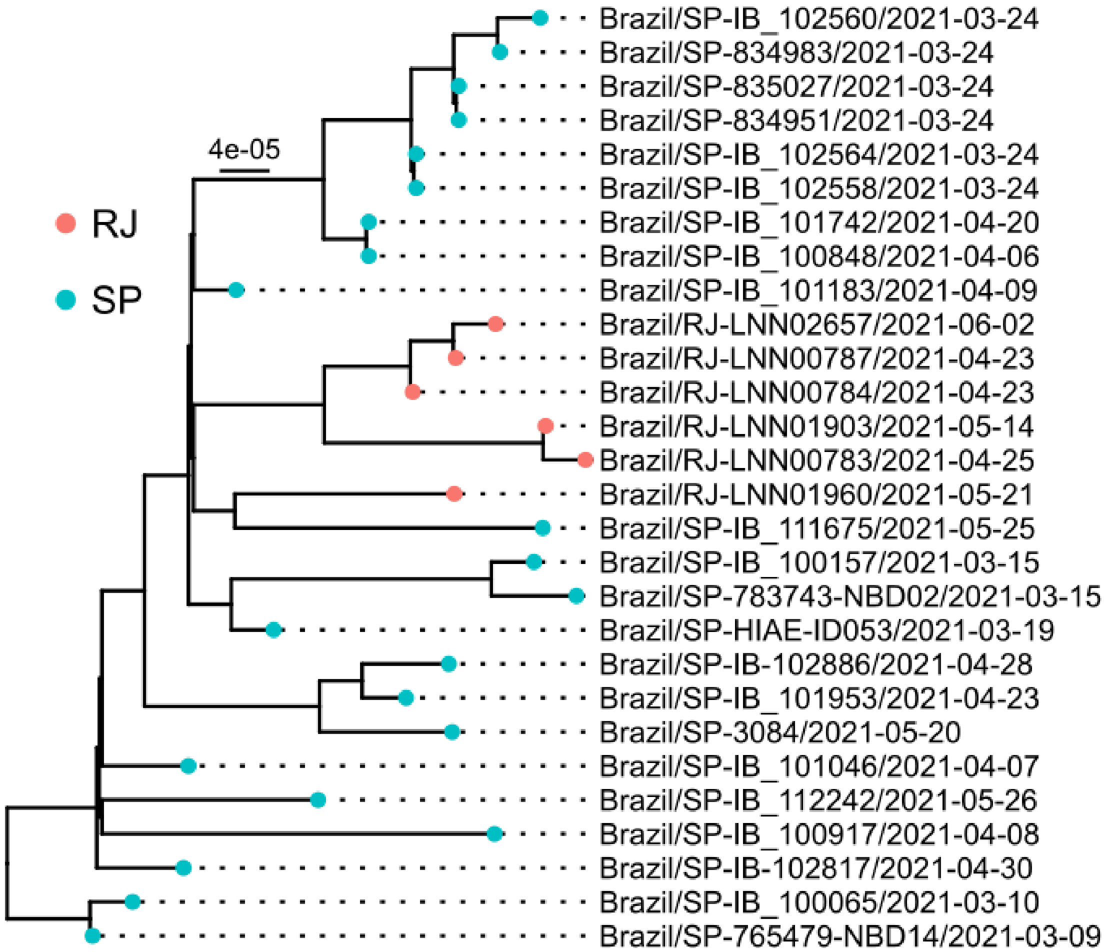

Figure S5. Maximum likelihood tree of the 28 SARS-CoV-2 genomes classified as P.5. Colors indicate the Brazilian state of origin of the sample.

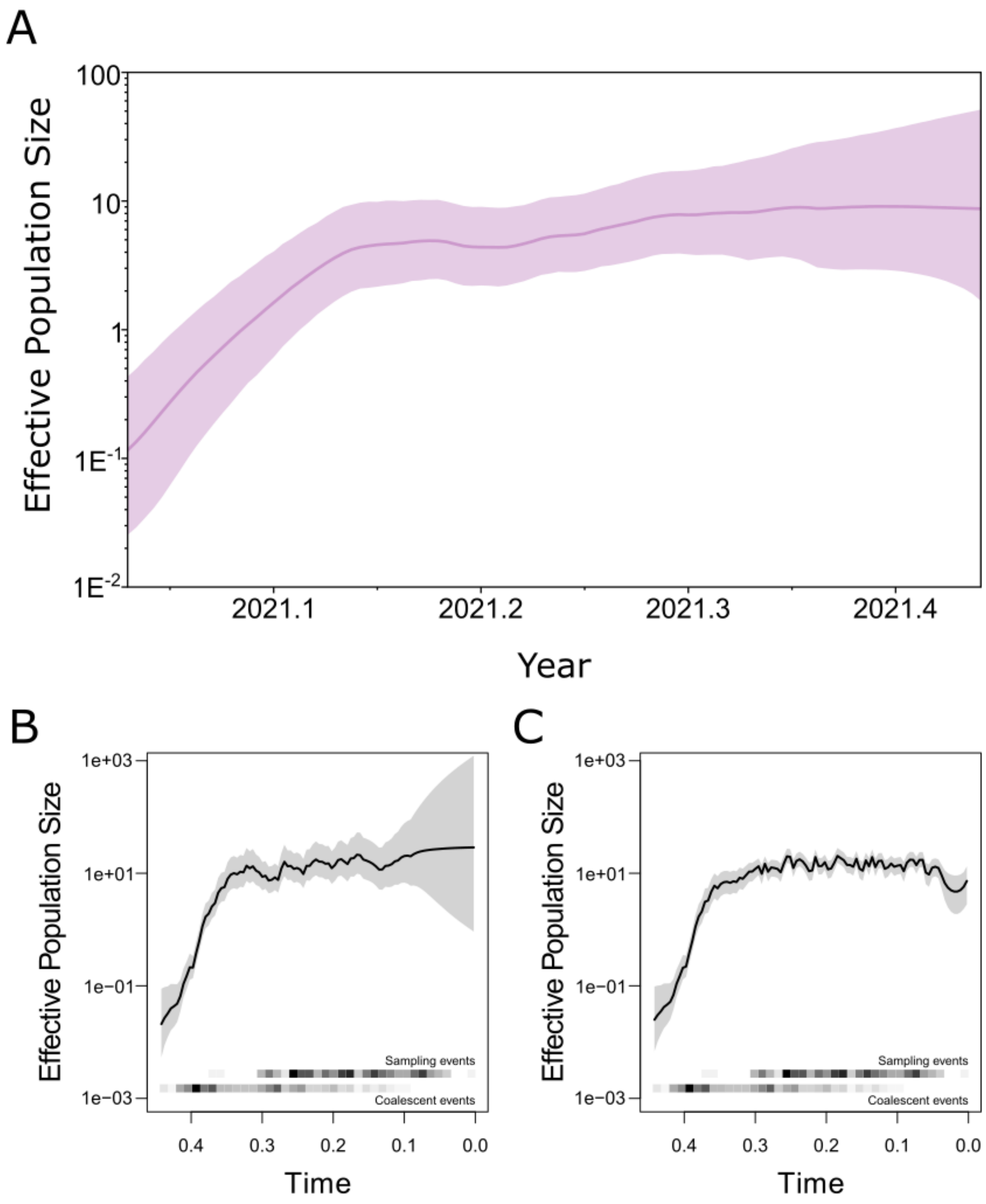

Figure S6. Effective population size of lineage P.1.2 across time inferred with different models. A) The GMRF Skyride population model available in BEAST was used. B-C) Bayesian Nonparametric Phylodynamic Reconstruction model was used without (B) and with (C) preferential sampling correction.

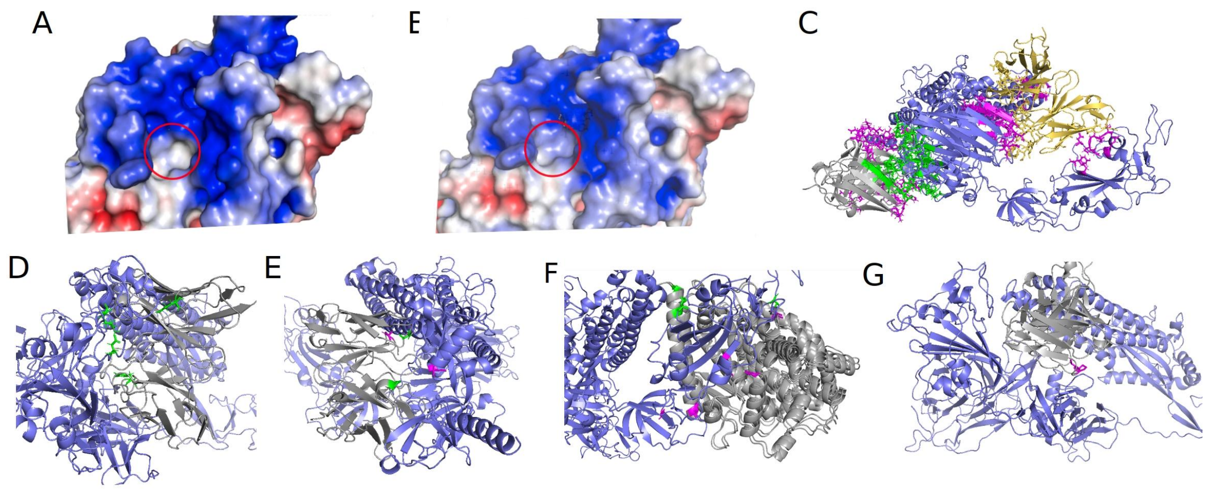

Figure S7. Comparison of P.1 and P.1.2 protein structures using receptor-ligand docking and affinity binding prediction. A-B) Electrostatic potential surface of P.1 and sub-clade P.1.2 structures. Red circle indicates the residuo 262 where the mutation (A262S) occurs. C-G) Receptor-ligand complexes for four interactions between Spike and neutralizing antibodies and one interaction between Spike and ACE2. In blue the receptor and the ligands in gray; P.1 exclusive residues are highlighted in pink and those exclusive from P.1.2 are colored in green. In (C), we projected the location of the antibody S1notRBD in P.1 in yellow, maintaining its location in P.1.2 as gray.

Table S1. Prevalence of COVID-19 cases in the macroregions in the state of Rio de Janeiro

Region	Median	IQR
Central	9.835030	6.050624
North	8.941276	4.812837
Northwest	7.548458	3.940123
South	7.248784	4.403857
Lowland Coastal	6.404055	4.471987
Metropolitan	5.035158	5.192506

Table S2. Comparison of age mortality among the phases.

Ages	Phases	Adjusted p-value (BH)
00-04	2nd and 3rd	0.003
05-09	1st and 3rd	0.034
10-19	1st and 2nd	0.032
10-19	2nd and 3rd	0.015
20-29	1st and 3rd	<2e-16
20-29	2nd and 3rd	<2e-16
30-39	1st and 3rd	<2e-16
30-39	2nd and 3rd	<2e-16
40-49	1st and 2nd	0.013
40-49	1st and 3rd	<2e-16
40-49	2nd and 3rd	<2e-16
50-59	1st and 3rd	<2e-16
50-59	2nd and 3rd	<2e-16
60-69	1st and 2nd	0.00014
60-69	1st and 3rd	2.2e-12
60-69	2nd and 3rd	1.2e-08
70-79	1st and 2nd	1.5e-05
70-79	1st and 3rd	0.0019
80-89	1st and 2nd	1.5e-05
80-89	2nd and 3rd	1.3e-06
90 and > 90	1st and 2nd	9.2e-09
90 and > 90	2nd and 3rd	4.6e-15

Table S3. Proportion of Genomes deposited in GISAID and number of cases from the state of Rio de Janeiro.

Month	No. Genomes Sequenced	No. Cases Confirmed	Proportion (%)
2020/03	45	7369	0.6107
2020/04	253	50535	0.5006
2020/05	54	60156	0.0898
2020/06	37	52331	0.0707
2020/07	93	53927	0.1724
2020/08	93	45997	0.2022
2020/09	54	41028	0.1316
2020/10	80	41157	0.1944
2020/11	71	92057	0.0771
2020/12	119	112478	0.1058
2021/01	162	70254	0.2306
2021/02	187	38291	0.4883
2021/03	433	96427	0.4490
2021/04	911	77517	1.1752
2021/05	913	74462	1.2261
2021/06	427	9248	4.6172

Table S5. Mutations present in more than 75% of sequences of each lineage deposited in the GISAID database, summarized by the Lineage Comparison tool available at https://outbreak.info/

Gene	B.1.1.33	B.1.1.28	P.1	P.1.2	P.2	P.5
ORF1a	-	-	S1188L	S1188L	-	-
ORF1a	-	-	-	-	-	T1637I
ORF1a	-	-	K1795Q	K1795Q	-	-
ORF1a	-	-	-	-	-	A3209V
ORF1a	-	-	-	-	L3468V	-
ORF1a	-	-	S3675K	S3675K	-	-
ORF1a	-	-	DEL3676/3678	DEL3676/3678	-	-
ORF1a	-	-	-	-	-	Q3729K
ORF1a	-	-	-	-	L3930F	-
ORF1a	-	-	-	-	-	P4337L
ORF1b	P314L	P314L	P314L	P314L	P314L	P314L
ORF1b	-	-	E1264D	E1264D	-	-
S	-	-	-	-	-	F2L
S	-	-	-	-	-	Q14K
S	-	-	L18F	L18F	-	-
S	-	-	T20N	T20N	-	-
S	-	-	P26S	P26S	-	-
S	-	-	-	-	-	T95I
S	-	-	D138I	D138I	-	-
S	-	-	R190S	R190S	-	-
S	-	-	K417T	K417T	-	-
S	-	-	E484K	E484K	E484K	E484Q
S	-	-	N501Y	N501Y	-	N501T
S	D614G	D614G	D614G	D614G	D614G	D614G
S	-	-	H655Y	H655Y	-	-
S	-	-	T1027I	T1027I	-	-
S	-	V1176F	V1176F	V1176F	V1176F	V1176F
ORF3a	-	-	S253P	S253P	-	-
ORF6	I33T	-	-	-	-	-

ORF8	-	-	E92K	E92K	-	-
N	-	-	P80R	P80R	-	-
N	-	-	-	-	A119S	-
N	R203K	R203K	R203K	R203K	R203K	R203K
N	G204R	G204R	G204R	G204R	G204R	G204R
N	-	-	-	-	-	G215V
N	-	-	-	-	M234I	-
N	I292T	-	-	-	-	-

Table S6. Energy comparison between P.1 and sub-clade P.1.2 models using FoldX tool

Criteria	P.1	Sub-clade P.1.2
BackHbond	-662.35	-654.28
SideHbond	-198.52	-185.18
Energy_vdwclash	65.19	76.6
Entropy_mainc	1763.32	1754.78
**Total**	373.6	395.76

Table S7. Number of contacts in common between P.1 and sub-clade P.1.2 in complex with the antibodies and ACE2

Ligand	# Common AAs	# AA P.1	# AA Sub-clade P.1.2	AA P1	AA Sub-clade P.1.2
S1-S2	19	1	2	ASP-428.A	GLU-516.A;SER-459.A
ACE2	23	3	1	PRO-561.A;THR-523.A;PRO-330.A;SER-469.A	
S1-Not RBD	0	28	20	[...]	SER-262.A;[...]
S1-RBD	25	1	2	TRP-353.A	ASP-571.A;THR-1006.A
S2	25	1	1	TYR-707.A	GLY-1124.A
